# Photocatalytic Properties of Eco-Friendly ZnO Nanostructures on 3D-Printed Polylactic Acid Scaffolds

**DOI:** 10.3390/nano11010168

**Published:** 2021-01-11

**Authors:** Maria Sevastaki, Vassilis M. Papadakis, Cosmin Romanitan, Mirela Petruta Suchea, George Kenanakis

**Affiliations:** 1 Institute of Electronic Structure and Laser, Foundation for Research & Technology-Hellas, N. Plastira 100, 70013 Heraklion, Greece; msevastaki@iesl.forth.gr (M.S.); billyp@iesl.forth.gr (V.M.P.); 2 Department of Chemistry, University of Crete, 71003 Heraklion, Greece; 3 Institute of Molecular Biology and Biotechnology, Foundation for Research & Technology-Hellas, N. Plastira 100, 70013 Crete, Greece; 4 National Institute for Research and Development in Microtechnologies (IMT-Bucharest), 1 26 A, Erou Iancu Nicolae Street, P.O. Box 38-160, 023573 Bucharest, Romania; cosmin.romanitan@imt.ro; 5 Center of Materials Technology and Photonics, Hellenic Mediterranean University, 71004 Crete, Greece

**Keywords:** green synthesis ZnO, natural Cu doping, eco-friendly photocatalytic materials, nanomaterials, paracetamol degradation

## Abstract

The present paper reports a novel approach for fabrication of eco-friendly ZnO nanoparticles onto three-dimensional (3D)-printed polylactic acid (PLA) scaffolds/structures. Several alcohol-based traditional Greek liquors were used to achieve the corrosion of metallic zinc collected from a typical galvanic anode to obtain photocatalytic active nanostructured ZnO, varying from water, to Greek “ouzo” and “raki”, and pure ethanol, in combination with “Baker’s ammonia” (ammonium bicarbonate), sold worldwide in every food store. The photocatalytic active ZnO nanostructures onto three-dimensional (3D)-printed PLA scaffolds were used to achieve the degradation of 50 ppm paracetamol in water, under UV irradiation. This study provides evidence that following the proposed low-cost, eco-friendly routes for the fabrication of large-scale photocatalysts, an almost 95% degradation of 50 ppm paracetamol in water can be achieved, making the obtained 3D ZnO/PLA structures excellent candidates for real life environmental applications. This is the first literature research report on a successful attempt of using this approach for the engineering of low-cost photocatalytic active elements for pharmaceutical contaminants in waters.

## 1. Introduction

Photocatalysis is a well-known technique with many applications in the degradation of organic pollutants [1]. Among other pollutants, unused or expired pharmaceuticals are commonly discharged into drains, and as a result, pharmaceutical compounds have recently been detected in surface, ground water, or even drinking water with many adverse impacts on both people and environment [2,3,4]. Worldwide research groups are working daily to find new ways to reduce, or even convert, pharmaceutical waste to non-toxic substances based on photocatalytic approaches. The scientific community makes a lot of effort to reduce this problem, taking into account the use of metal oxides. Zinc oxide (ZnO) is a suitable material both for scientific as well as industrial applications. Its wide direct band gap of 3.37 eV [5] and the large exciton binding energy of 60 meV are the characteristics that make it a unique candidate for such applications. Another important characteristic is the wide range of morphological diversity of ZnO in nanoscale, varying from nanoparticles, to nanorods, nanowires, nanopins and nanotubes, etc. [6,7,8,9,10,11,12].

It is worth mentioning that ZnO can be grown in the form of nano- or micro-structures, following chemical solution approaches, at mild conditions and low temperature. For instance, a lot of research works cite the fabrication of ZnO nanostructures using Zn salts in aqueous solutions, such as highly oriented ZnO nanowires [13], while several other morphologies have been reported, such as whisker arrays using solvothermal approaches at low temperatures [14,15], nanorods [16,17,18], and nanofibers [19], etc. With the exception of Zn salts, metallic Zn has also been used in precursor solutions in order to eliminate the use of reagents and to synthesize ZnO. For example, flower-like nanostructures can be synthesized using metallic Zn by employing a NaCl solution corrosion-based approach [19] on metallic Zn.

For the last several years, quite a lot of research groups have tried to find/follow “green” approaches (synthesis using naturally available reducing agents) for the synthesis of nanostructured materials [20,21], and to use environmentally friendly and non-toxic precursors in order to reduce consumption of high purity reagents with high environmental footprints and costs, compared to conventional chemical methods [5,21,22,23,24,25].

This article reports on the fabrication of ZnO nano- and micro- structures using metallic Zn recycled from a typical galvanic anode that is typically used in any building and everyday use hydraulic systems. Moreover, in order to evolve the approach of Yan et al. [19], to an environmentally friendly one, several alcohol-based traditional Greek spirits were used to achieve the corrosion of metallic Zn to ZnO, varying from water, to Greek “ouzo” and “raki”, and pure ethanol, in combination with “Baker’s ammonia” (ammonium bicarbonate) [26,27,28], which is sold worldwide in every food store. Greek “ouzo” and “raki” are two of the most common alcoholic drinks, and they can also be found in ethnic stores all over the world. They are comparable with several other worldwide consumed spirits [29,30,31].

As already stated [10], three-dimensional (3D) printing provides an effective way to fabricate large-scale photocatalytic devices with a high surface-to-volume ratio [32,33]. This study tries to combine the environmentally friendly chemical synthesis of ZnO nanostructures based on recycled Zn anodes with environment-friendly biocompatible polylactic acid (PLA) 3D-printed scaffolds, in order to fabricate photocatalytic devices with enhanced performance in the removal of pharmaceutical pollutants from water. We provide evidence that the produced 3D-printed photocatalysts provide a photocatalytic efficiency of ~95% against 50 ppm paracetamol in water, making them excellent candidates for real life applications. This is the first literature research report on a successful attempt of using this approach for the engineering of low-cost photocatalytic active elements for pharmaceutical contaminants in waters.

## 2. Experimental Details

### 2.1. Production of 3D-Printed Scaffolds

**Figure 1:**
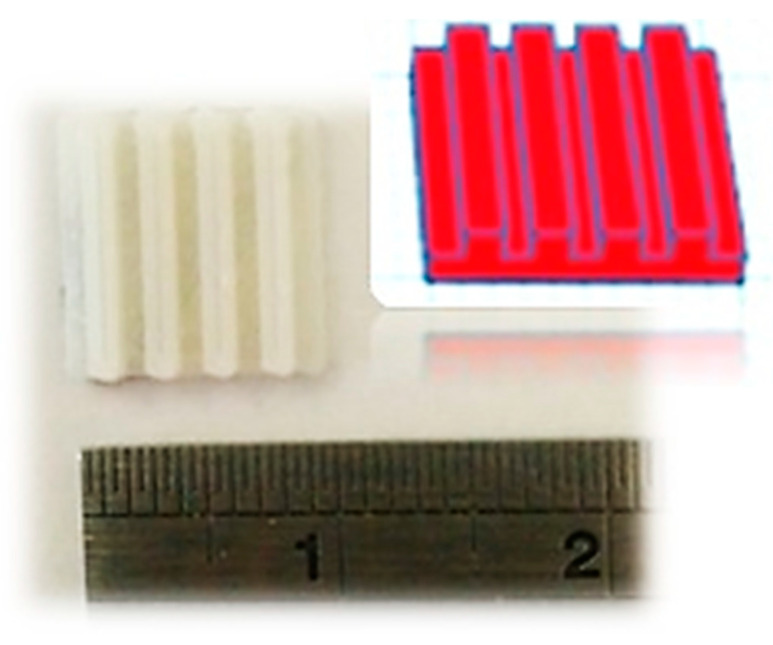
Representative image of the 3D-printed structures/scaffolds.

Based on the need of photocatalysis for a large active surface availability, first, “Tinkercad”, a free online 3D modeling program from Autodesk Inc. (Mill Valley, CA, USA), was used to design the loophole rectangular-shaped structure (10 mm × 10 mm × 2 mm) presented in Figure 1. Afterwards, a dual-extrusion FDM-type 3D printer (MakerBot Replicator 2X; MakerBot Industries, Brooklyn, NY, USA) was used for the fabrication of 3D structures/scaffolds, using a commercially available PLA filament (MakerBot Industries, Brooklyn, NY, USA).

At this this point, it should be noted that PLA was chosen over a huge variety of commercially available polymeric filaments, since it is a petroleum-free biodegradable plastic derived from renewable sources, made mainly from the fermentation of corn starch [28], and also because it was found to release less total volatile organic compounds (VOCs) than acrylonitrile butadiene styrene (ABS) or other fused filaments [29,30].

The fused deposition modeling (FMD) process of building a solid object involves heating of the filament and pushing it out layer-by-layer through a heated (220 °C) nozzle (0.4 mm inner diameter) onto a heated surface (60 °C), via a computer controlled three-axis positioning system (with a spatial resolution of approximately 100 μm in z-axis and 11 μm in x and y). Following the approach described above, 3D-printed samples were fabricated layer-by-layer, with each layer fixed at 0.2 mm, and the filling factor at 100%, indicating that all samples are fully packed. The geometry, along with the dimensions of the 3D-printed scaffolds used in this work, is presented in Figure 1.

### 2.2. Synthesis of the Photocatalytic Nanostructure

The printed PLA structures were used as substrates for further deposition of nanostructured ZnO obtained from recycled metallic Zn anode following a general approach reported elsewhere [20].

**Figure 2:**
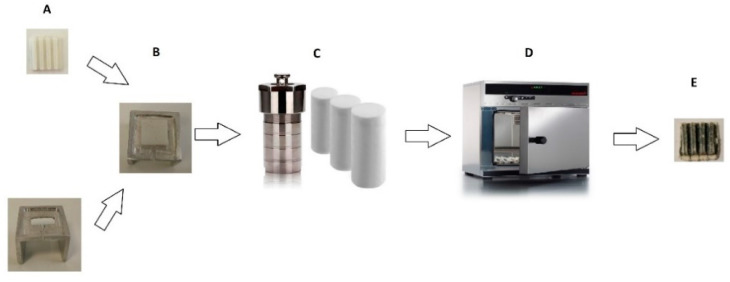
Illustration of nanostructured ZnO synthesis steps onto 3D-printed substrate. (**A**) 3D-printed polylactic acid (PLA) substrate; (**B**) Metal base with substrate on top; (**C**) Stainless steel (ss) autoclaves; (**D**) Oven at 200 °C for 2 h; **E.** Final ZnO-coated PLA 3D-printed scaffolds.

The PLA substrate was placed on a metal base with the upper side facing down in a beaker, as shown in Figure 2, to prepare the chemical composition and to avoid precipitation of filings of Zn metal. In the stainless steel (SS 316) autoclaves 50 mL solutions (Eth, H_2_O, raki and ouzo, respectively) with 74.3675 mg metallic Zn and 70.101 mg baking NH_3_ were added. The stainless steel autoclaves were heated at 200 °C for 2 h in an oven. After that, the substrates were removed and dried at 60 °C in air.

### 2.3. Characterization and Photocatalytic Experiments

#### 2.3.1. Scanning Electron Microscopy–Energy-Dispersive X-Ray Spectroscopy

The surface morphology of the ZnO-coated 3D printed structures was studied using a field emission scanning electron microscope (FE-SEM, JEOL JSM-7000F; JEOL Ltd., Tokyo, Japan), equipped with an energy-dispersive X-ray spectroscopy (EDX) analyzer (Oxford Instruments, High Wycombe, UK). The microscopy characterization was done in high vacuum (HV) using ~10 nm Au/Pd coated samples. The EDX analysis was reported as an average over at least 3 analyzed areas per sample.

#### 2.3.2. X-ray Diffraction

X-ray diffraction (XRD) experimental analysis was used to determine the crystal structure of the prepared ZnO-coated 3D-printed structures by using a Rigaku (RINT 2000) (Rigaku, Tokyo, Japan) diffractometer with Cu Kα (λ = 1.5406 Å) X-rays for 2θ = 30.00–70.00° and a step time of 0.02°/s.

#### 2.3.3. Raman Spectroscopy Studies

Raman spectroscopy measurements were performed at room temperature using a Horiba LabRAM HR Evolution confocal micro-spectrometer (HORIBA FRANCE SAS, Longjumeau, France), in backscattering geometry (180°), equipped with an air-cooled solid-state laser (HORIBA FRANCE SAS, Longjumeau, France) operating at 532 nm with 100 mW output power. The laser beam was focused on the samples using a 10× Olympus microscope objective (OLYMPUS corporation, Tokyo, Japan) (numerical aperture of 0.25), providing ~14 mW of power on each sample. Raman spectra over the 100–700 cm^−1^ wavenumber range (with an exposure time of 5 s and 3 accumulations) were collected by a Peltier cooled CCD (1024 × 256 pixels) detector at −60 °C, with a resolution better than 1cm^−1^, which was achieved thanks to an 1800 grooves/mm grating and an 800 mm focal length. Test measurements were carried out using different optical configurations, exposure times, beam power, and accumulations in order to obtain sufficiently informative spectra using a confocal hole of 100 μm, taking care to avoid alteration of the sample, while the high spatial resolution allowed us to carefully verify the sample homogeneity. The wavelength scale was calibrated using a Silicon standard (Silchem H×andelsgesellschaft mbH, Freiberg, Germany) (520.7 cm^−1^), and the acquired spectra were compared with scientific published data and reference databases, such as Horiba LabSpec 6 (HORIBA FRANCE SAS, Longjumeau, France).

#### 2.3.4. Photocatalytic Efficiency Measurements

The photocatalytic activity of the 3D-printed samples was studied by means of the reduction of 50 ppm aqueous solution of paracetamol, a well-known pharmaceutical product that has been used as an organic model to probe the photocatalytic performance of photocatalysts [4,5,7,8]. The investigated samples were placed in a custom-made quartz cell, and the whole setup (cell + solution + sample) was illuminated for up to 60 min using a UV lamp centered at 365 nm (Philips HPK 125 W) (msscientific Chromatographie-Handel GmbH, Berlin, Germany) with a light intensity of ~6.0 mW/cm^2^. The concentration of paracetamol (degradation) was monitored by UV–Vis spectroscopy in absorption mode (absorption at λ_max_, 665 nm), using a K-MAC SV2100 spectrophotometer (K-MAC, Daejeon, Korea) over the wavelength range of 220–800 nm. In this way, UV–Vis absorption data were collected at 0, 10, 20, and 30 min, while the quantification of the paracetamol degradation (and hence the remaining paracetamol concentration) was estimated by calculation of the area below the main paracetamol peak in the range of 220–320 nm. Additional blank experiments (photolysis) without a catalyst were also performed as well as paracetamol adsorption experiments in the dark. To ensure repeatability, the photocatalysis experiments were performed at least 5 times.

## 3. Results and Discussion

### 3.1. SEM and EDX Analyses

**Figure 3:**
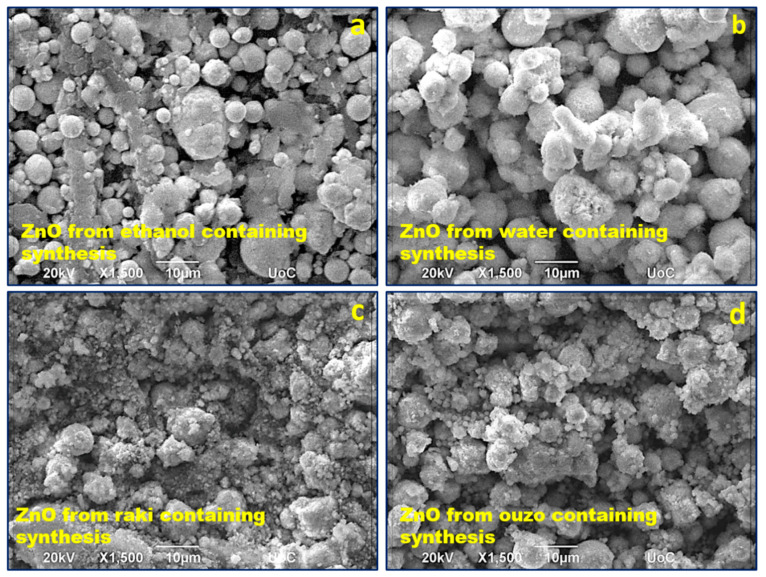
SEM imaging of ZnO synthesized from (**a**) zinc powder and baking NH_3_ in ethanol, (**b**) zinc powder and baking NH_3_ in water, (**c**) zinc powder and baking NH_3_ in ouzo, and (**d**) zinc powder and baking NH_3_ in raki.

In the case of these eco-friendly ZnO depositions, the obtained ZnO coating structuring consisted of nanostructured spherical particles with a broad range of diameters from tens of nm up to ~10 μm. Figure 3a–d shows FE-SEM micrographs presenting how the coating structuring differed for these samples prepared using the environmentally friendly way (recycled Zn metal, baking NH_3_) with different solvents: ethanol, water, ouzo, and raki, respectively. From the SEM images, one can observe the strong influence of the solvent on ZnO coating morphology that can be related to its degree of adherence to the PLA substrate as well as changes in the active surface. It can be observed that the ZnO synthesized only in water led to the largest particle size. The particles were “fluffy” agglomerations of smaller grains. The synthesis using ethanol solvent led to smaller particles and rather compact, larger agglomerations. The use of the two traditional Greek spirits as solvents seemed to determine a dramatic decrease in particle size. The studies on the effect of using the ouzo and raki solvents are ongoing, and their results will be the subject of a further scientific report.

**Figure 4:**
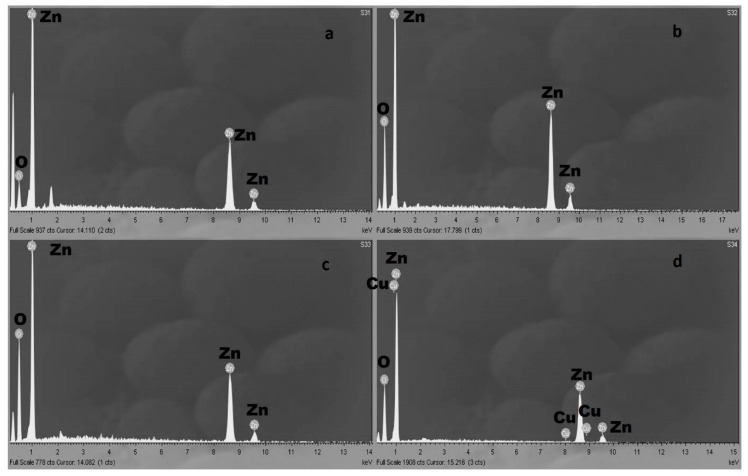
(**a**) A typical EDX spectrum of ZnO synthesized in ethanol, (**b**) a typical EDX spectrum of ZnO synthesized in water, (**c**) a typical EDX spectrum of ZnO synthesized in raki, and (**d**) a typical EDX spectrum of ZnO synthesized in ouzo.

The EDX analysis of all the ZnO coatings compositions showed the presence of pure, stoichiometric ZnO except to the material synthesized using ouzo. In the case of ouzo containing synthesis, the ZnO coatings EDX analysis show the presence of Cu element. Cu presence can be attributed to anise, an herb which was used for the different smell and taste of ouzo and Cu containing as supported in literature [27]. Figure 4 presents typical EDX spectra of the obtained ZnO coatings.

### 3.2. XRD Analysis

**Figure 5:**
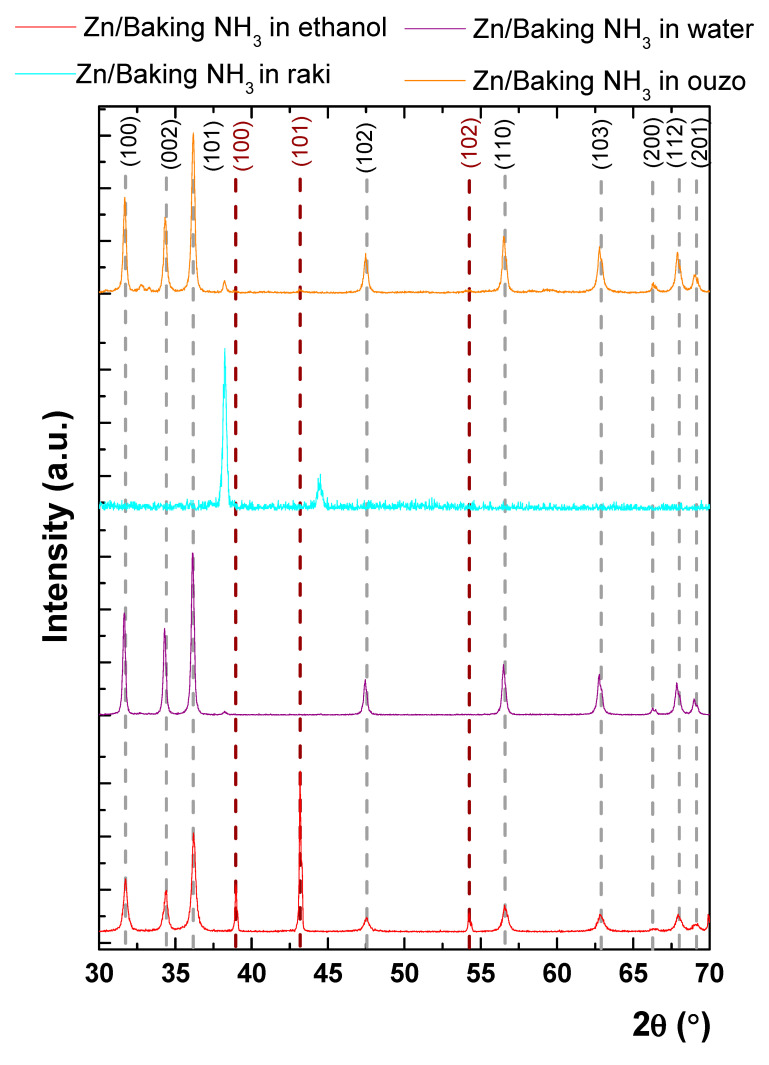
XRD for ZnO nanostructured coatings synthesized starting from zinc powder and baking NH_3_ in ethanol (red line), water (violet), raki (blue), and ouzo (orange). The dashed grey line was used to show the position of ZnO diffraction peaks, while the brown one was used for metallic Zn.

Figure 5 presents the XRD patterns obtained for the investigated samples as follows: red (Zn/baking NH_3_ in ethanol), violet (Zn/baking NH_3_ in water), blue (Zn/baking NH_3_ in raki), and orange (Zn/baking NH_3_ in ouzo).

The peak indexing was made using the ICDD—International Center for Diffraction Data—database. One can observe that the samples presented typical diffraction peaks for ZnO according to card no. 01-1136, belonging to P63/mmc (186) spatial group, excluding the Zn/baking NH_3_ in raki. The position of the diffraction peaks corresponding to ZnO was indicated with a grey dashed line at 2θ: 31.76°, 34.54°, 36.29°, 47.39°, 56.50°, 62.89°, 66.27°, 67.78°, and 69.17°, which correspond to the following Miller indices: (100), (002), (101), (102), (110), (103), (200), (112), and (201), respectively. Whereas ZnO synthesized in NH_3_, water, or ouzo presented the typical diffraction peaks for ZnO, and in the case of the one synthesized in raki, the diffraction peaks for ZnO were absent. Accordingly, the obtained ZnO had the lattice parameters a = b = 0.32 nm and c = 0.52 nm. Furthermore, in the case of the ZnO/baking NH_3_ in ethanol sample, the other diffraction peaks can be indexed as metallic Zn (card no.04-0831)—highlighted with a brown line at 39.11°, 43.15°, and 54.26° that corresponds to (100), (101), and (102) reflections.

The obtained lattice parameters for Zn in ethanol were a = b = 0.26 nm, while c = 4.94 nm, values that are corresponding to metallic Zn. Additionally, in the case of the ethanol solvent, the coexistence of the metallic Zn and ZnO phases with a percentage of 36% for the first phase can be observed. The percentage of phases was evaluated with the RIR (reference intensity ratio) method using PDXL software developed by Rigaku. Furthermore, in the case of water solvent, an almost perfect oxidation of metallic Zn took place, with only a small feature of Zn at 38.24° being observed, while the percentage of metallic Zn dramatically decreased to below 1%. A small quantity of metallic Zn was also observed in the case of ouzo solvent, where the percentage of metallic Zn was slightly greater than 1%. For raki solvent use, crystalline ZnO was not present and all diffraction peaks could be assigned to metallic Zn. Moreover, for raki, the diffraction peaks were shifted with respect to the standard position at 38.24° and 44.27°, respectively, meaning, according to Bragg’s law [34,35], a tensile strain on (100) direction and a compressive one on (101) direction. In the case of use of ouzo solvent, a tensile strain on (100) direction was observed. A possible explanation for the observed strain may arise from the incorporation of the oxygen into zinc lattice, but without forming ZnO bonds in the first case. In this context, the formation of crystalline ZnO was strongly influenced by the used solvent, which led to different oxidation reactions of metallic Zn. Regarding the crystal quality of the formed ZnO, the Scherrer equation was employed. It is well-known that the diffraction peak width, *β* can be related with the size of the crystalline domains, *τ* in the following way:

(1) $$\tau =\;\frac{k\lambda }{\beta \cos\theta }$$

where k is the shape factor taken as 0.9, taking into account the spherical form of the grains, as highlighted by SEM micrographs, λ = 0.154 nm is the wavelength of the X-rays and θ is the angular position. It was observed that the mean crystallite size calculated on ZnO (101) reflection ranged from 36.3 nm (for both ethanol and water) to 37.9 nm for ZnO synthetized in ouzo. Hence, a better crystal quality was obtained in ouzo, which can be further ascribed with a smaller dislocation density in the ZnO lattice. The XRD results indicate that the kind of used solvent does not affect the interplanar distance of ZnO, but it leads to a slight modification of the mean crystallite size.

### 3.3. Raman Spectroscopy Analysis

Raman scattering is the inelastic scattering of photons by phonons. For UV–visible photons the direct photon–phonon coupling is weak and their interaction is mainly mediated by electrons via an electron–radiation Hamiltonian, exciting the material into an intermediate (virtual) state, with the creation of an electron–hole pair. The electron–hole pair is then scattered into another state by emitting (or absorbing) a phonon via the electron–phonon interaction Hamiltonian. Finally, it recombines radiatively, emitting the scattered photon with a lower or higher energy, while the electronic state of the material remains unchanged [36,37]. So, as photons interact with the lattice, Raman scattering measurements can provide information regarding both the vibrational modes of the structure and the electronic properties of the material [36]. The phonons that can do the scattering can be defined based on the structure of the crystal, and as the fundamental Raman selection rule states, only phonons near Γ (wavevector q~0) are measured. As shown by XRD characterization, the eco-synthesized ZnO coatings had a wurtzite crystalline structure. One of the most frequent ZnO crystallization forms is the wurtzite structure, a hexagonal lattice characterized by two interpenetrating sub-lattices of Zn^2+^ and O^2−^ ions such that each Zn ion is surrounded by a tetrahedron of oxygen ions and vice versa. This arrangement is characterized by polar symmetry along the hexagon vertical axis (c axis). Wurtzite structure of ZnO crystal has C_6v_ symmetry. There are 4 atoms in the hexagonal unit cell leading to 12 phonon branches, 9 optical, and 3 acoustic. The nine optical phonons are divided into one A_1_ branch (Raman and IR active), one doubly degenerate E_1_ branch (Raman and IR active), two doubly degenerate E_2_ branches (Raman active only), and two inactive B_1_ branches. Thus, there are four Raman active phonons at the center of the Brillouin zone. In fact, the Raman spectrum of bulk ZnO presented six first-order peaks according to [38,39,40,41], not four as the number of active modes. The two most intense peaks were associated to the E_2_ modes, the first at ~100 cm^−1^ (named E_2_ low), dominated by the vibrations of the metallic Zn sub-lattice, and the second at 437–438 cm^−1^ (named E_2_ high), which involves mostly the oxygen atoms. The two modes are defined non-polar modes. On the contrary, the A_1_ and E_1_ phonons, which are oxygen-dominated, are polar modes. Consequently, the two A_1_ and E_1_ modes split into LO and TO components. For this reason, the associated Raman peaks become four, i.e., A_1_(TO) at 378–380 cm^−1^, A_1_(LO) at 574–579 cm^−1^, E_1(_TO) at 409–413 cm^−1^, and E_1_(LO) at 583–591 cm^−1^, which together with the two E_2_ modes leads to the six detectable Raman peaks [38,39,40,41].

**Figure 6:**
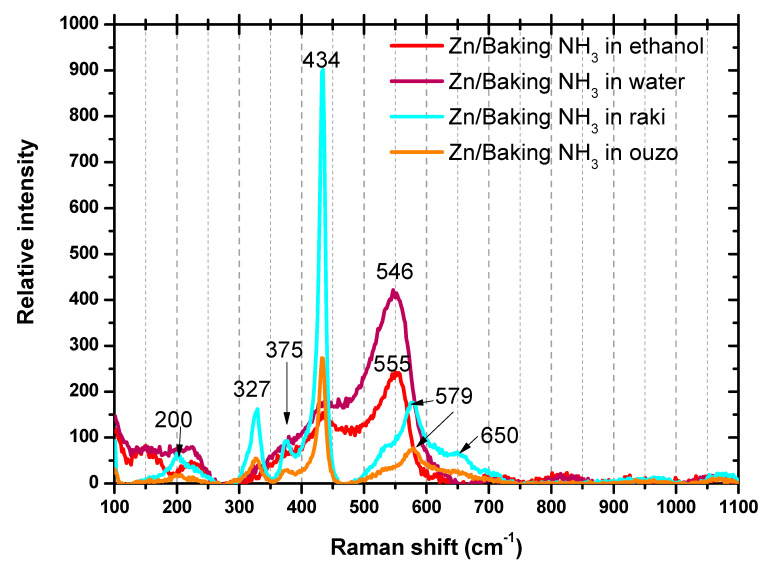
Raman spectra of the ZnO coatings synthesized from eco-friendly precursor materials.

As shown in Figure 6 for the eco-friendly ZnO coatings, the Raman spectra show the presence of the eigen modes of E_2_ high and A_1_(LO) of ZnO for the materials synthesized in raki and ouzo located at 434 cm^−1^ and 579 cm^−1^, respectively, additionally 2E_2_low and E_2_high-E_2_low modes occurred at 200 cm^−1^ and 327 cm^−1^, respectively. For the ethanol and water ZnO samples, wide absorption bands of ~225 cm^−1^ and 546 cm^−1^~555 cm^−1^ emerged. The ~225 cm^−1^ bands may be attributed to the presence of interstitial zinc (Zn_i_). The 546 cm^−1^~555 cm^−1^ peaks can be generated by the oxygen vacancy defect (V_o_). Since the characteristic vibration peaks associated with oxygen vacancy (V_o_) defect at 579 cm^−1^ overlap with the eigenmode of A_1_(LO), it is very difficult to confirm whether the strong vibration absorption peaks at 579 cm^−1^ were caused by A_1_(LO). Further studies and correlation with XRD observations are ongoing to elucidate the raki and ouzo synthesized ZnO nanomaterials.

### 3.4. Photocatalysis

The reduction of paracetamol in aqueous solution was evaluated by the photocatalytic activity of the environmentally friendly structures under UV-A light. Photolytic removal of the pharmaceutical product in the absence of any photocatalyst was negligible, underlining the necessity of the catalysts. In addition, in order to eliminate the possibility of paracetamol removal by adsorption on the catalysts, the samples were placed at the bottom of the reactor under dark conditions and in contact with the paracetamol for 40 min, during which time equilibrium of adsorption–desorption was achieved. In all cases, removal was insignificant (less than 3%), pointing to the fact that the reduction of the paracetamol should be attributed to a pure photocatalytic procedure.

**Figure 7:**
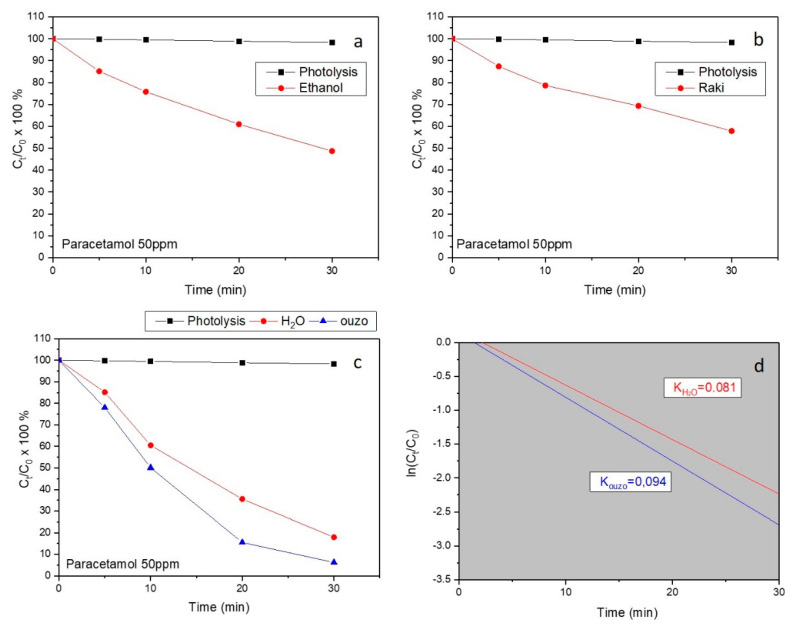
The percentage of paracetamol decomposition over the ZnO-based 3D-printed samples synthesized with eco-friendly solvents under UV-A irradiation, vs. irradiation time. (**a**) Ethanol. (**b**) Raki. (**c**) Two different solvents are presented H_2_O and ouzo (red solid circles and blue solid triangles, respectively). (**d**) The apparent rate constants (k) for water and ouzo cases. For comparison reasons, the photolysis curve (black solid squares) is also presented.

An example of the typical decrease of paracetamol concentration (50 ppm) in the presence of ZnO samples synthesized in ethanol, raki, H_2_O, and ouzo under UV-A light irradiation are presented in Figure 7a–c. It is noticeable that using both kinds of samples (H_2_O and ouzo) ~80% and ~95% paracetamol (50 ppm) degradation, respectively, was successful. Ethanol and raki synthesized samples led to much lower photocatalytic activities (~50 and ~40% of 50 ppm paracetamol). For comparison reasons, the photolysis curve (no catalyst present) is also displayed in the graphic. According to the photolysis (black) curves in Figure 7a–c, the concentration of paracetamol remained almost constant during ~30 min irradiation, indicating that the photolysis of paracetamol was almost negligible.

In addition, the apparent rate constant (k) has been calculated as the basic kinetic parameter for the comparison of photocatalytic activities, which was fitted by the equation ln (C_t_/C_0_) = −kt, where k is apparent rate constant, C_t_ is the concentration of paracetamol, and C_0_ is the initial concentration of paracetamol. It should be noted that the adjusted R-square statistic varied from 0.88375 to 0.99124, indicating that the model used for the determination of the apparent rate constant (k) is adequate. The good linear fit of equation ln (C_t_/C_0_) = −kt, shown in Figure 7d, confirms that the photodegradation for two different samples (synthesized in H2O and ouzo) photocatalysts follows first-order kinetics. Finally, we observe faster degradation at sample synthesized in ouzo and it may be due to the different morphology caused by the increase in active surface area of the material. Further studies regarding the photocatalytic activity correlation with the materials structuring are ongoing.

## 4. Conclusions

To summarize, the successful synthesis of environmentally friendly ZnO structured coatings utilizing materials that we encounter in everyday life as precursors, such as zinc filings, baking NH_3_, H_2_O, raki, and ouzo, is reported in this first literature research report on a successful attempt of using this approach for the engineering of low-cost photocatalytic active elements for pharmaceutical contaminants in waters. Structure and morphology of ZnO coatings strongly depend on the kind of solvent used. Using ouzo as a solvent, natural Cu doping can be achieved. This may be the reason for the enhanced photocatalytic activity in paracetamol decomposition observed in the experiments. Further studies to understand and control the natural Cu doping will be performed. The ZnO synthesis was performed onto 3D-printed PLA structures used as a substrate. The ZnO-coated PLA 3D-printed structures were studied for their photocatalytic activity for decomposition of paracetamol as pharmaceutical common pollutant, and the results were incredible. Almost 95% degradation of 50 ppm paracetamol in 30 min was achieved for the ZnO synthesized in ouzo-coated sample. This has excellent promise for achieving ecologic and biocompatible photocatalytic materials at low cost, and for providing a practical way for the eventual fabrication of large-scale, environmentally friendly photocatalysts suitable for water purification and biologic applications.

## Data Availability

The data are available upon request from the corresponding authors.
